# Copper Amalgam—Discrimination in Its Use Necessary to Success

**Published:** 1892-12

**Authors:** Geo. H. Weagant


ARTICLE VII.

### COPPER AMALGAM—DISCRIMINATION IN ITS USE, NECESSARY TO SUCCESS.

---

I do not know exactly what relations the different metals bear to each other in the state called fusion, whether the minute infinitesimal particles of each metal remain intact and distinct from each other; but, whatever fusion is, I think that the same condition exists in an amalgam. There may be particles of free copper in copper amalgam, and in the coarser grades I think there are; but their surfaces must be covered with an alloy composed of copper and mercury fused into each other. It is reasonable to suppose this coating of alloy has a perceptible depth, and the finer those particles are the more complete the fusion, and if we follow it up we can imagine a state of affairs when this fusion goes clean through the minute particles of copper, and then there would be no free copper contained in the mass. We can cover a piece of copper completely with mercury by rubbing it in; so, by grinding copper amalgam and breaking up the minute particles of copper and rubbing in the mercury upon them we get more complete fusion. But, better still, if the copper, in the first place, be precipitated in as fine a state as possible upon the mercury, we have a more thorough fusion still, for we know that copper precipitate. in a nascent state, amalgamates or fuses with mercury.

The precipitate from a strong solution of a cupric salt is coarser grained than from a weak solution. If the solution was diluted with an equal bulk of water, the precipitate would be twice as fine; the weaker the solution the finer the precipitate, and the more complete the fusion between the copper and the mercury, in short, the more thorough the amalgamation. I agree with Dr. Ames and Dr. Custer that the copper amalgam must be thoroughly amalgamated and of fine grain to give the best results. I go further and repeat what I have always maintained, that it must be thoroughly clean and free from all kinds of impurities, especially oxide of copper or mercury or any other metal. If we overheat it or allow it to become wet or damp while mixing, it becomes more or less oxidized and therefore unclean. I attribute the discoloration of tooth substance by copper amalgam fillings to impurities in the material.

I received a letter not long ago from a prominent American dentist asking me what was my theory concerning the wasting of copper amalgam fillings. He says, "we know copper amalgam will save teeth; now, we want to know what will save the filling." I was obliged to confess I had no theory to advance. But I have been experimenting with the material for a number of years. I have made a great many experiments both in the mouth and out of it, and have kept a record of fillings put in and notes of the various methods employed and peculiarities observed. The results of my observations, I fear, do not amount to a great deal. They have certainly not led to the adoption of a theory. But I have learned to avoid many things which invite failure, and to recognize indications which promise success. I have noticed that failures are more likely to occur in some localities than in others, for instance: copper amalgam fillings placed in approximal cavities extending over the grinding surface of molars and bicuspids, are more apt to fail than all others. Large crown and contour fillings in molars come next. These fail from the wasting or cupping described before. Buccal fillings very rarely fail unless they extend to the grinding surface, when "cupping" generally takes place. Small crown and fissure fillings seldom fail, and approximal fillings, anterior and posterior, which do not reach the grinding surface, are almost invariably successful. The most permanent and successful I find are fillings in those shallow groove-like cavities on the palato-cervical and linguo-cervical surface of molars and bicuspids, and sometimes of canines and incisors.

In my own practice I have very few failures, and I dare say it is because I use copper amalgam very cautiously. I never employ it where other amalgams would do as well, nor where experience teaches me it was likely to fail. I have never considered it to be a substitute for other materials, but rather a material to use where I knew anything else would fail. I have not begun to have the number of failures in my work that other dentists report in theirs, and I have seen more failures in fillings made by other dentists than in my own, but I think that is because they use it too indiscriminately. In fact some have told me they have used it altogether to the exclusion of every other kind, and I have no doubt it is due to this fact that it has proved so disastrous in many hands. Dr. Trueman says that he "considers copper amalgam judiciously used, a valuable addition to our list of tooth-saving materials. It does not, however, supplant the alloy amalgams." He is disposed to think "that in the long run, those who use it most cautiously will appreciate it most highly."

I have never seen recurrence of decay around a copper amalgam filling, except in those small shallow buccal cavities in molars and bicuspids at the margin of the gum, where we find a disintergation and softening of enamel. I think the fault in these cases lies in not cutting enough of the enamel away in the first place. I would not blame the amalgam, as another material would have done no better—certainly not alloy amalgam. It is in those very same shallow buccal cavities where the use of copper amalgam is specially indicated; just there where it exhibits, in strong contrast to other amalgams, its peculiar unshrinking qualities. Even the small amount which good alloy amalgams shrink would be quite sufficient to cause it to "drop out."

Of course, in speaking of failures, one would not include those cases where recurrence of decay was caused by the accidental fracture of the tooth at the margin of the cavity. My experience has been that there is less liability of marginal fractures where copper amalgam is employed than where we use an alloy amalgam, and I account for it in this way: There being no shrinkage with copper amalgam, the enamel is better supported, and consequently not so apt to break. With some alloys we know there is considerable shrinkage, and a space is left under a thin portion of enamel inviting fracture sooner or later.

In mixing copper amalgam or preparing it for the cavity, if it is too soft it will result in disintegration and softening of the filling. Neither must it be used hard and dry. See that it works with a smooth plastic finish under the burnisher. If it is dry and granular under a burnisher, it will result in failure. It is not difficult to determine the proper plasticity to give best results, as when it works smoothest and easiest is when it is right; and when it is properly mixed it certainly is a very pleasant material to work.

Do not insert the copper amalgam after it has begun to set, for your filling will fail. After it has begun to set it should not be reheated until thoroughly hard.

Dr. Ames argues that we heat and triturate it too much. Dr. Barnes and Dr. Osmun contend that we do not heat and grind it enough. It is my opinion we heat it too much and triturate it too little. When I say heat it too much, I do not mean too often, but with too high a temperature. Copper amalgam should be heated slowly over a small flame, care being taken not to raise the temperature too high. If care is taken, it does not matter how often we heat it and allow it to get hard again; the oftener the better; but I think that part of it is the manufacturer's business to attend to.

I would like to say a word in reference to mortars. It is important to have a good one. Some of the small porcelain mortars to be found at the depots are worse than useless—smooth inside, with a pestel also smooth and too small to grasp. Some of the larger glass and wedgewood mortars are better, but some of the pestles are too large. The mortar I have found to give the best satisfaction is a ground glass mortar and pestle made by Fletcher of England.—GEO. H. WEAGANT, *Dominion Dental Journal.*

